# Complete heart block in dengue complicating management of shock due to both bleeding and leakage: a case report

**DOI:** 10.1186/s13104-015-1036-9

**Published:** 2015-03-04

**Authors:** Mitrakrishnan Rayno Navinan, Jevon Yudhishdran, Sandamalee Herath, Isurujith Liyanage, Tharshana Kugadas, Damith Kumara, Aruna Kulatunga

**Affiliations:** National Hospital of Sri Lanka, Colombo, Sri Lanka

**Keywords:** Dengue, Myocarditis, Heart block, Arrhythmia, Electrocardiograms, Expanded dengue syndrome

## Abstract

**Background:**

The spread of Dengue virus infection is reaching pandemic proportions. Dengue is usually dreaded for causing shock due to capillary leakage. However the clinical spectrum of dengue is vast and the newly incorporated expanded dengue syndrome introduces a wide range of presentations that are rarely observed and appreciated but nevertheless have the potential to cause significant morbidity and even mortality. Cardiac involvement in dengue is one such example.

**Case presentation:**

A 26 year old South-Asian female presented in a state of haemodynamic shock with a history of fever and use of non-steroidal anti inflammatory drugs. Dengue was suspected clinically and later confirmed. Following stabilization and while still in the febrile phase the patient developed bradycardia with dynamic electrocardiogram changes which evolved into complete heart block. However there was no circulatory compromise. Clinical picture was further complicated by the development of dengue haemorhaghic fever and cautious fluid resuscitation was carried out in correlation to clinical and haematological parameters. Impaired coagulation profile necessitated administration of activated factor seven on the backdrop of low platelets and bleeding. Cardiac pacing could be avoided due to maintenance of vitals within acceptable parameters.

**Conclusion:**

Expanded dengue syndrome should be given greater appreciation as not all may be benign. Cardiovascular system involvement in dengue has the potential to cause significant morbidity and mortality. Careful interpretation of clinical parameters will help in the institution of the appropriate management and help avoid unnecessary invasive interventions. Screening of dengue patients with timely electrocardiographs would be useful to detect cardiac involvement. Guidance on managing atypical manifestations of dengue expanded syndrome should available to help clinicians dictate treatment.

## Background

Dengue is the most common mosquito borne illness. Having affected more than 100 countries worldwide with a global at risk population exceeding 40%, dengue viral infection is truly reaching pandemic proportions. Thus with increased prevalence a rising trend is seen in both morbidity and mortality [1]. In a dengue endemic country, when patients present with fever along with the complaints of well recognized symptoms, a clinical diagnosis of dengue viral infection is made and treatment initiated while awaiting confirmation, in view of a possible progression to variable categories of shock due to capillary leakage. Atypical manifestations of dengue however are not adequately appreciated and recognized. Single organ involvement is sometimes the only recognizable insult seen in dengue, and this phenomenon is classified as the expanded dengue syndrome [2]. Cardiac involvement is one such situation. The complications that arise due to cardiac involvement are numerous, and range from arrhythmias which can be benign to potentially fatal [3], such as fulminant myocarditis [4]. The lack of specificity in the patterns of elicited symptoms make it a challenge to categorically identify its involvement without a high level of suspicion especially when by itself it is an uncommon phenomenon [5]. With the rising trend in disease burden, the spectrum of clinical manifestations seen have also increased, resulting in a greater recognition of these atypical situations, including cardiac manifestations, necessitating a better understanding of these atypical situations as they can cause significant morbidity and mortality, stressing the need for early identification which can help in treatment and intervention [6-8]. In this paper, we present a case of a young female who sustained myocardial insult with development of bradycardia with progressive ST and T segmental changes finally evolving into complete heart block in the background of shock attributable to both haemorrhage and later capillary leakage, presenting a uniquely complicated clinical picture.

## Case presentation

**Figure 1:**
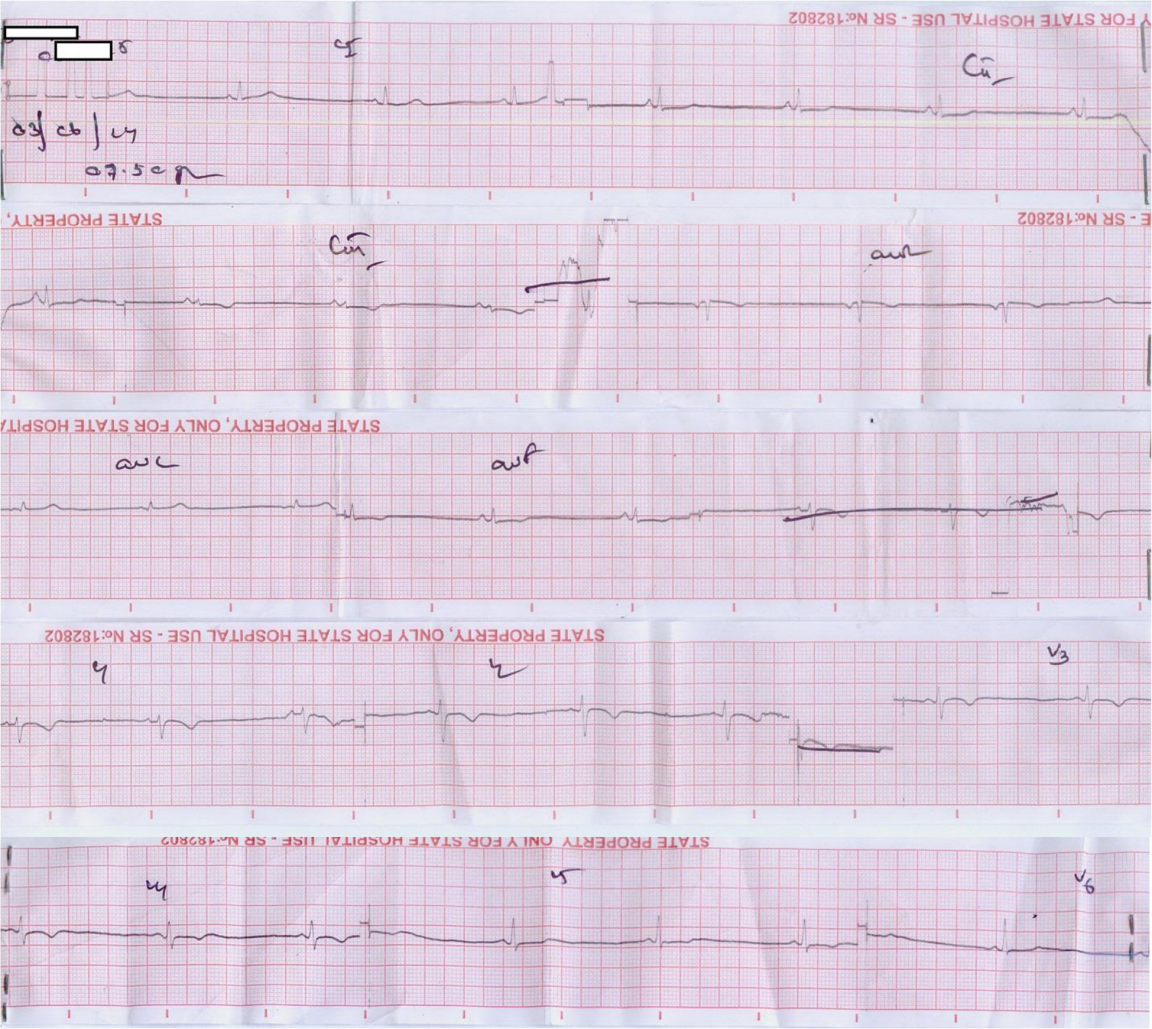
**Preliminary ECG.** The initial electrocardiogram shows bradycardia with inverted T waves from V_2_-V_5_.

**Figure 2:**
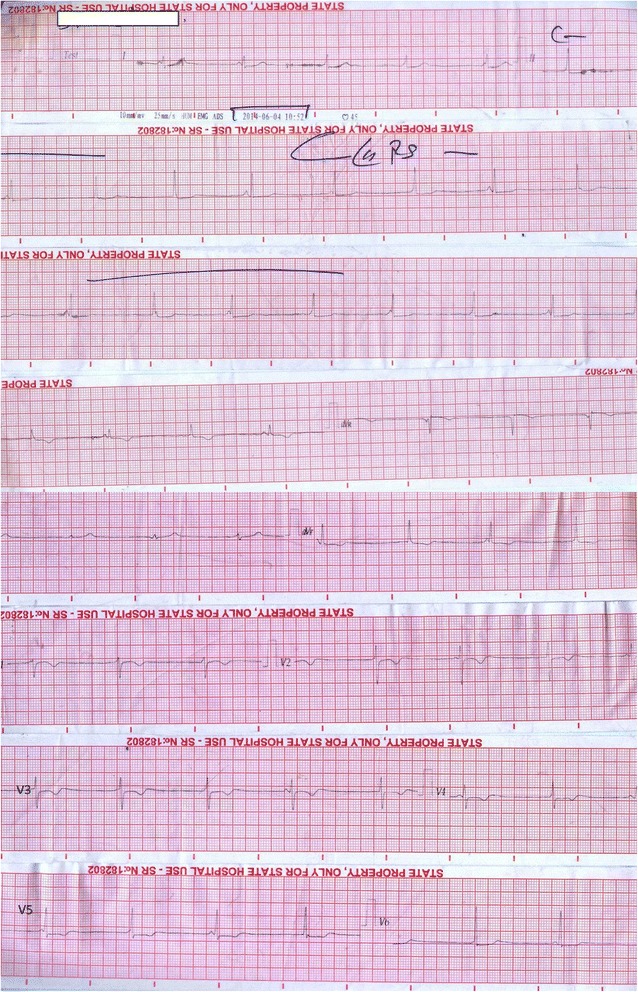
**Progressive changes.** The subsequent electrocardiogram shows P/QRS wave dissociation with complete heart block and a junctional rhythm.

A 26 year old South-Asian female, having irregular menstrual cycles but was otherwise healthy presented with fever to the accident & emergency unit with severe generalized myalgia and arthralgia. Clinical assessment revealed a tachycardia with a pulse rate of 108 b.p.m and an initial blood pressure of 90/50 mmHg, indicating shock. Clinical picture favored dengue fever with the classic repertoire of symptoms. Since the patient had taken mefenamic acid, a non steroidal anti-inflammatory agent (NSAID) for myalgia for a few days, her complaint of fever for only one day at presentation suggested an erroneous timeline and a late presentation. Rest of the systemic examination was normal and clinically there was no evidence of leakage of fluid in the chest and abdomen on presentation. She was started immediately on an intravenous infusion of 0.9% saline. Preliminary whole blood analysis demonstrated a low haematocrit of 32% with a reduced haemoglobin of 10.4 g/dL (11–17) and a platelet count of 173 × 10 ^9^/L (150–450). Despite fluid replacement her blood pressure declined to 80/40 mmHg with development of shock together with a reduction of the haematocrit to 29%. At this stage haemorrhagic shock was suspected on a background of NSAID usage. She was transfused with cross matched packed red cells urgently and stabilization of the haemodynamic parameters were noted with a borderline but stable blood pressure of 100/60 mmHg. Haematocrit also stabilized at 35%. An ultra sound scan failed to reveal either leakage of fluid into pleural cavity or the presence of ascitic fluid and intra-abdominal heaemorrhage. However the patient suffered heavy per vaginal bleeding which continued. She was observed in a high dependency setting and 8 hours after admission and clinical stabilization, she developed bradycardia, which was confirmed on electrocardiogram (ECG). In addition to sinus bradycardia, the ECG showed T wave inversions from V2- V5 (Figure 1). Cardiac insult due to dengue virus infection was suspected, although creatine kinase-MB was normal and Troponin I remained negative. ECG was done serially to monitor the progression and on the second day of hospitalization revealed the occurrence of complete heart block with atrio-ventricular dissociation and a junctional rhythm with a short P-R interval (Figure 2). Despite this, the blood pressure remained within acceptable safe limits. In view of satisfactory peripheral perfusion a clinical decision was made not to insert a temporary pacemaker, but to continue close observation for possible haemodynamic compromise occurring due to arrhythmia or worsening due to its presence.

Primary dengue virus infection was confirmed by the presence of positive IgM antibodies with negative IgG antibodies on day 2 of hospitalization. Platelet counts on day 2 of hospitalsation was 157 ×10^9^/L(150–450), but haematocrit value started to rise to 42 and then 45 within a few hours, and clinical examination revealed a right sided pleural effusion which was confirmed on sonographic imaging with the presence of pleural fluid and additionally fluid in the hepatorenal pouch. Fluid resuscitation and replacement was carried out in accordance with the national guidelines on dengue management. Serum magnesium levels were within reference range, with a value of 2.07 mg/dL (1.58-2.55). Serum sodium and potassium values were within reference throughout her stay. Initial serum ionized calcium levels were found to be low normal with a value of 1.14 mmol/L(1.12-1.32) Subsequent ionized calcium levels were found reduced to a value of 1 mmol/L (1.12-1.32). In the background of ECG changes, hypocalcemia was corrected cautiously with intravenous calcium gluconate and the electrolytes were monitored regularly, though the cardiac rhythm remained unchanged. Post correction serum ionized calcium was 1.17 mmol/L and later 1.22 mmol/L (1.12-1.32). Two dimensional echocardiogram revealed an ejection fraction of 50% with normal valves, normal sized atria and ventricles with no systolic wall motion abnormalities though myocardial depression was observed. Liver function tests revealed elevated enzymes. Aspartate transaminase and alanine transaminase were 141U/L (<40) and 119 U/L (<40) respectively and later rose to the highest recorded value of 320 U/L(<40) and 187U/L (<40) respectively. Gamma GT was also elevated at 347.5 U/L (7–50). Total protein was normal with a value of 62.4 g/L (60–85) but albumin was marginally reduced to 30.9 g/L (35–54). The patient remained clinically stable but the persistence of bleeding prompted a blood picture analysis to identify and exclude features of disseminated intravascular coagulation (DIC). Blood picture revealed moderately low platelets and reactive lymphocytes but no features of DIC. However thromboelastography revealed hypocoagulation, platelet deficiency and fibrinogen deficiency. In light of low platelets (lowest nadir of 10x109/L) and continuous per vaginal bleeding 4 units of factor VII complex was given. Normal sinus rhythm was noted on day 5 of admission, though she remained bradycardic till the time of discharge from hospital.

The bleeding ceased and platelet counts rose gradually. The patient made good clinical recovery with the pleural effusion subsiding and vaginal bleeding ceasing. ECG showed resolution upon review two weeks later.

## Discussion

Cardiac involvement in dengue can result in a spectrum of presentations, some benign and inadvertently detected due to the continuous monitoring that dengue patients are subjected to frequently and may not raise much concern. But others can have deleterious effects on both the patient due to its direct effect or worsen the clinical state since the basis of management in dengue virus infection is resuscitation with fluids.

Arrhythmias are the commonest abnormality found in cardiac involvement in dengue and sinus tachycardia is probably the most frequently seen phenomenon. Occurring either during the febrile phase or when developing Dengue haemorrhaghic fever with fluid leakage. This may or may not be due to cardiac involvement. Other manifestations include T wave abnormalities, ST segment depressions and elevation, sinus pauses, ectopics that are either atrial or ventricular in origin, ventricular trigeminy, atrial fibrillation, heart blocks such as first-degree block and Mobitz type I second-degree AV block, bundle branch blocks [3,6,9-13] and rarely complete atrio-ventricular dissociation [14-16]. Bradycardia is more commonly seen in defervescence and convalescence, due to parasympathetic activity. It has the potential to precede other arrhythmias like complete AV dissociation or VPC, thus it’s detection should prompt more intense monitoring [2,17,18].

The pathophysiological basis for arrhythmias in dengue are variable, the favored theory is that it occurs due to viral myocarditis even in the absence of structural heart disease [9]. The myocarditis per se is brought about due to the viral tropism for the heart. The host immune response counters the virus by the release of cytokines, a phenomenon known as “cytokine storm”, which due to viral heart involvement causes inflammatory insult resulting in the loss of integrity of both structure and function thus causing arrhythmia [19,20]. Localized insult due to minute bleeding involving the sino-atrial node, atrio-ventriular node or within its close vicinity can also cause transient conduction abnormalities. Similarly transient abnormalities can also be indicative of a functional, than a structural insult. This can be due to altered autonomic tone, adenosine metabolism or even electrolyte imbalance that may occur in dengue viral fever [21]. Hypocalcemia is commonly seen even after correction for low albumin levels, especially when in leakage with resulting dengue haemorrhagic fever [22]. Hypocalcemia is known to cause lengthening of the ST segment and prolonging the QT interval while sparing the T wave. However rare reports detail hypocalcemia as a possible cause for ST depressions with even dyskinesia of the heart musculature demonstrable on angiogram [23]. Prolonged QT poses a risk of additional arrhythmias including atrial arrhythmias, torsades de pointes and even ventricular arrhythmias [24]. Hyponatraemia is also known to occur in dengue [25] and is up to ten times more prevalent compared to that of non-dengue viral febrile illness, especially when patients proceed to shock in dengue haemorrhaghic fever (DHF). The phenomenon is attributable to salt deficiency, renal loss, redistribution into cells due Na-K pump dysfunction and excess water due to reduced renal excretion [26]. Very low sodium levels have been known to be associated with arrhythmias including first degree, second degree heart blocks and even complete heart block [27]. Hypokalemia has also been a documented feature, it is hypothesized that low potassium occurs due to the redistribution of potassium into cells or renal loss secondary to that of renal tubular injury and even to the catecholamine rise seen due to the viral infection [28]. Both serum potassium and sodium levels are comparatively lower during the state of capillary leakage (DHF) and when the level of shock is greater [29]. Thus, electrolyte imbalance which has the propensity to occur in dengue can theoretically cause arrhythmias. Thus stressing the importance of both checking for and correcting these imbalances when clinically indicated.

Drugs are notorious in causing cardiac insult and arrhythmias. In dengue, fluid resuscitation is the only known and required treatment in most instances. But in the presence of prior drug history one should also clinically assess its possible impact on the cardiac system. The concept of drug induced arrhythmias are vast and are outside the spectrum of discussion of this case report. But in consideration to non-steroidal anti-inflammatory drugs, which is unfortunately a commonly abused class of drug, has the potential to cause a lot of side effects and cardiac insult has also been observed. NSAIDS has been reported to increase the occurrence of arrhythmias such as atrial fibrillation [30]. Additionally in background of heart disease it can even precipitate heart failure [31]. Our patient was an infrequent user of mefenamic acid, an NSAID used for menstrual pain. The wide abuse of this drug and the clinical range of complications it can cause should prompt clinicians to inquire its recent use, when facing febrile illness that has the potential to cause bleeding manifestations.

Unlike typically observed patterns the occurrence of bradycardia and subsequent complete heart block preceded that of leakage (DHF) in our patient and occurred in the febrile phase. However as mentioned in the Comprehensive Guidelines for Prevention and Control of Dengue and Dengue Haemorrhagic Fever [2], complete heart block did occur after bradycardia, stressing the importance that the simple process of monitoring heart rate may help to detect possible sequalae that are expected. Although hypocalcemia was present, the deficit was mild and correction did not revert the arrhythmia to sinus rhythm. NSAIDS were a risk factor in our patient, but the observed arrhythmia did not correlate to what is commonly seen. The use of NSAIDS however did possibly contribute towards the increased risk of bleeding that was seen in our patient. The observed bradycardia with T wave abnormalities and third degree heart block is possibly attributable to direct myocardial involvement, though biochemical markers failed to elicit objective cardiac insult. Two dimensional echo findings however favored myocarditis. Our patient most likely had myocarditis due to dengue causing complete heart block. Though shock occurred the clinical situation and laboratory parameters favored actual occult haemorrhage as the cause for shock, which responded to blood transfusion and fluid resuscitation. Cardiac pacing was not required as haemodynamic parameters remained satisfactory following the blood transfusions and resuscitation with intravenous fluids despite the persistence of complete heart block.

## Conclusion

Cardiac involvement in dengue should have greater recognition as manifestations may not always be benign. Arrhythmias being the commonest should be actively sought for as it can be an early indicator for myocarditis, which should not be missed as fluid resuscitation being the primary treatment of dengue hinges heavily on the stability of the cardiovascular system. Clinical and laboratory parameters should be correlated and attempts made to correct the exact cause of the insult before active interventions such as cardiac pacing is considered. This highlights the point that though there maybe compromise in cardiac stability definitive intervention may only be required when haemodynamic compromise persists despite adequate resuscitation with fluids and blood volume restoration. Guidance however is required in a more definitive manner as a clear protocol will help clinicians treat patients with greater confidence ensuring good outcome.

## Consent

Written informed consent was obtained from the patient for publication of this Case Report and any accompanying images. A copy of the written consent is available for review by the Editor-in-Chief of this journal.

## Abbreviations

DHF: Dengue haemorrhaghic fever
NSAID: Non steroidal anti inflammatory drugs
ECG: Electrocardiogram

## References

[1] WHO. Dengue and severe dengue: Fact sheet N°117. WHO media centre; 2014 [updated March 2014]; Available from: http://www.who.int/mediacentre/factsheets/fs117/en/.

[2] World-Health-Organization (2011). Comprehensive Guideleines For the Prevention and Control of Dengue and Dengue Haemorrhagic Fever: Revised and expanded edition.

[3] La-Orkhun V, Supachokchaiwattana P, Lertsapcharoen P, Khongphatthanayothin A (2011). Spectrum of cardiac rhythm abnormalities and heart rate variability during the convalescent stage of dengue virus infection: a Holter study. Ann Trop Paediatr.

[4] Lee CH, Teo C, Low AF (2009). Fulminant dengue myocarditis masquerading as acute myocardial infarction. Int J Cardiol.

[5] Pesaro AE, D’Amico E, Aranha LF (2007). Dengue: cardiac manifestations and implications in antithrombotic treatment. Arq Bras Cardiol.

[6] Saldarriaga GC, Roncancio G, González N, Fortich F (2013). Manifestaciones cardiacas del dengue: Reporte de una serie de casos durante la epidemia colombiana de 2010. Revista Colombiana de Cardiología..

[7] Ron-Guerrero CS, Ron-Magaña AL (2011). Dengue fatal: reporte de cuatro casos en Nayarit, México. Medicina Interna de México.

[8] Nimmagadda SS, Mahabala C, Boloor A, Raghuram PM, Nayak UA (2014). Atypical manifestations of Dengue Fever (DF) - Where do we stand today?. J Clin Diagn Res.

[9] Horta Veloso H, Ferreira Junior JA, de Paiva JMB, Faria Honorio J, Junqueira Bellei NC, de Paola AAV (2003). Acute atrial fibrillation during dengue hemorrhagic fever. Braz J Infect Dis.

[10] Matthias AT, Indrakumar J, Gunatilake SB (2014). Ventricular trigeminy in a patient with serologically confirmed dengue haemorrhagic fever. Int Arch Med..

[11] Kularatne SA, Pathirage MM, Kumarasiri PV, Gunasena S, Mahindawanse SI (2007). Cardiac complications of a dengue fever outbreak in Sri Lanka, 2005. Trans R Soc Trop Med Hyg.

[12] Zea D, Foley K, Carey J (2014). Myocarditis in a traveler returning from the dominican republic: an unusual presentation of dengue Fever. Am J Trop Med Hyg.

[13] Jindal A, Shivpuri D (2013). Heart involvement in dengue viral infection in children. Crit Care Med.

[14] Lim SMS, Hoo FK, Sulaiman WAW (2014). A case of dengue hemorrhagic fever with myocarditis and complete heart block. RMJ.

[15] Kaushik JS, Gupta P, Rajpal S, Bhatt S (2010). Spontaneous resolution of sinoatrial exit block and atrioventricular dissociation in a child with dengue fever. Singapore Med J.

[16] Martha JW (2009). Total atrioventricular block due to Dengue myocarditis. J Cardiovasc Electrophysiol.

[17] Promphan W, Sopontammarak S, Pruekprasert P, Kajornwattanakul W, Kongpattanayothin A (2004). Dengue myocarditis. Southeast Asian J Trop Med Public Health.

[18] Carter R, Hinojosa-Laborde C, Convertino VA (2014). Heart rate variability in patients being treated for dengue viral infection: new insights from mathematical correction of heart rate. Front Physiol..

[19] Salgado DM, Eltit JM, Mansfield K, Panqueba C, Castro D, Vega MR (2010). Heart and skeletal muscle are targets of dengue virus infection. Pediatr Infect Dis J.

[20] Tisoncik JR, Korth MJ, Simmons CP, Farrar J, Martin TR, Katze MG (2012). Into the eye of the cytokine storm. Microbiol Mol Biol Rev.

[21] Sharma JK, Zaheer S (2014). Variable atrio-ventricular block in dengue fever. J Indian Acad Clin Med.

[22] PPS. Department of Health Revised Dengue Clinical Case Management Guidelines 2011. 2011; Available from: http://www.doh.gov.ph/sites/default/files/revised_%20dengue_clinical_management_guidelines.pdf.

[23] RuDusky BM (2001). ECG abnormalities associated with hypocalcemia. Chest.

[24] Nijjer S, Ghosh AK, Dubrey SW. Hypocalcaemia, long QT interval and atrial arrhythmias. BMJ case reports. 2010;2010:bcr0820092216. Epub 2010/01/01. 10.1136/bcr.08.2009.2216 PMC3029862 22242081

[25] Singhi S, Kissoon N, Bansal A (2007). Dengue e dengue hemorrágico: aspectos do manejo na unidade de terapia intensiva. J Pediatr (Rio J)..

[26] Mekmullica J, Suwanphatra A, Thienpaitoon H, Chansongsakul T, Cherdkiatkul T, Pancharoen C (2005). Serum and urine sodium levels in dengue patients. Southeast Asian J Trop Med Public Health.

[27] Mouallem M, Friedman E, Shemesh Y, Mayan H, Pauzner R, Farfel Z (1991). Cardiac conduction defects associated with hyponatremia. Clin Cardiol.

[28] Kayal AK, Goswami M, Das M, Jain R (2013). Clinical and biochemical spectrum of hypokalemic paralysis in North: East India. Ann Indian Acad Neurol.

[29] Lumpaopong A, Kaewplang P, Watanaveeradej V, Thirakhupt P, Chamnanvanakij S, Srisuwan K (2010). Electrolyte disturbances and abnormal urine analysis in children with dengue infection. Southeast Asian J Trop Med Public Health.

[30] Granier M, Massin F, Pasquie JL (2013). Pro- and anti-arrhythmic effects of anti-inflammatory drugs. Antiinflamm Antiallergy Agents Med Chem.

[31] Feenstra J, Heerdink ER, Grobbee DE, Stricker BH (2002). Association of nonsteroidal anti-inflammatory drugs with first occurrence of heart failure and with relapsing heart failure: the Rotterdam Study. Arch Intern Med.

